# TP-064, a potent and selective small molecule inhibitor of PRMT4 for multiple myeloma

**DOI:** 10.18632/oncotarget.24883

**Published:** 2018-04-06

**Authors:** Kazuhide Nakayama, Magdalena M. Szewczyk, Carlo dela Sena, Hong Wu, Aiping Dong, Hong Zeng, Fengling Li, Renato Ferreira de Freitas, Mohammad S. Eram, Matthieu Schapira, Yuji Baba, Mihoko Kunitomo, Douglas R. Cary, Michiko Tawada, Akihiro Ohashi, Yasuhiro Imaeda, Kumar Singh Saikatendu, Charles E. Grimshaw, Masoud Vedadi, Cheryl H. Arrowsmith, Dalia Barsyte-Lovejoy, Atsushi Kiba, Daisuke Tomita, Peter J. Brown

**Affiliations:** ^1^ Oncology Drug Discovery Unit, Pharmaceutical Research Division, Takeda Pharmaceutical Company Limited, Fujisawa, Kanagawa 251-8555, Japan; ^2^ Structural Genomics Consortium, University of Toronto, Toronto, Ontario M5G 1L7, Canada; ^3^ Department of Pharmacology and Toxicology, University of Toronto, Toronto, Ontario M5S 1A8, Canada; ^4^ Medicinal Chemistry Research Laboratory, Pharmaceutical Research Division, Takeda Pharmaceutical Company Limited, Fujisawa, Kanagawa 251-8555, Japan; ^5^ Structiural Biology, Takeda California Inc., 10410 Science Center Drive, San Diego, CA 92121, USA; ^6^ Enzymology and Biophysical Chemistry, Takeda California Inc., 10410 Science Center Drive, San Diego, CA 92121, USA; ^7^ Princess Margaret Cancer Centre and Department of Medical Biophysics, University of Toronto, Toronto, Ontario M5G 2M9, Canada

**Keywords:** PRMT4, small molecule inhibitor, TP-064, crystal structure, multiple myeloma

## Abstract

Protein arginine methyltransferase (PRMT) 4 (also known as coactivator-associated arginine methyltransferase 1; CARM1) is involved in a variety of biological processes and is considered as a candidate oncogene owing to its overexpression in several types of cancer. Selective PRMT4 inhibitors are useful tools for clarifying the molecular events regulated by PRMT4 and for validating PRMT4 as a therapeutic target. Here, we report the discovery of TP-064, a potent, selective, and cell-active chemical probe of human PRMT4 and its co-crystal structure with PRMT4. TP-064 inhibited the methyltransferase activity of PRMT4 with high potency (half-maximal inhibitory concentration, IC_50_ < 10 nM) and selectivity over other PRMT family proteins, and reduced arginine dimethylation of the PRMT4 substrates BRG1-associated factor 155 (BAF155; IC_50_= 340 ± 30 nM) and Mediator complex subunit 12 (MED12; IC_50_ = 43 ± 10 nM). TP-064 treatment inhibited the proliferation of a subset of multiple myeloma cell lines, with affected cells arrested in G1 phase of the cell cycle. TP-064 and its negative control (TP-064N) will be valuable tools to further investigate the biology of PRMT4 and the therapeutic potential of PRMT4 inhibition.

## INTRODUCTION

Protein arginine methyltransferases (PRMTs) catalyze arginine methylation of proteins, which involves the transfer of the methyl group of S-adenosyl-L-methionine (SAM) to the terminal guanidino nitrogens of arginine. This reaction can give rise to three types of methylarginine species including monomethylarginine (MMA), symmetric dimethylarginine (SDMA), and asymmetric dimethylarginine (ADMA). PRMTs are classified according to their methylation products as Type I (PRMT1, 2, 3, 4, 6, and 8, which can convert arginine to MMA and ADMA), Type II (PRMT5 and 9, which produce MMA and SDMA), and Type III (PRMT7, which generates MMA only) [1]. Arginine methylation influences target interactions with other proteins and modulates their physiological functions [2].

PRMT4, also known as coactivator-associated arginine methyltransferase 1, is a Type I PRMT that methylates arginines 17 and 26 of histone H3 [3] as well as non-histone proteins involved in a variety of biological processes including transcriptional activation [4], RNA splicing [5], cell cycle regulation [6], DNA damage response [7], and cell differentiation [8]. PRMT4 is dysregulated in several diseases and has been linked to breast [6], prostate [9], and colorectal cancer [10] and positively regulates transcriptional activators including Wnt/β-catenin in colorectal cancer [11], estrogen receptor-α in breast cancer [12], Runt-related transcription factor 1 in myeloid leukemia [13], and the Switch/sucrose non-fermentable chromatin remodeling complex in breast cancer [14]. These reports suggest that PRMT4 is a potential therapeutic target in certain types of cancer.

Potent, selective, and cell-active small molecule PRMT4 inhibitors can be useful tools for clarifying the molecular events regulated by protein arginine methylation and for validating PRMT4 as a therapeutic target. A number of PRMT4 inhibitors have recently been reported [15]. For example, potent and selective PRMT4 inhibitors were developed that did not exhibit cellular activity [16, 17], while a cell-active inhibitor of Type I PRMTs that is not selective for PRMT4 was also reported [18]. Since Type I PRMTs (except for PRMT8) are ubiquitously expressed [19], improving the selectivity for PRMT4 can avoid adverse events caused by other Type I PRMT inhibitors. Recently a potent, selective, and cell-active inhibitor has been reported, EZM2302, which shows activity in preclinical models of multiple myeloma [20].

Here we report the development of a potent and selective PRMT4 inhibitor with high cellular activity. The co-crystal structure of PRMT4 in complex with N-methyl-N-((2-(1-(2-(methylamino)ethyl)piperidin-4-yl)pyridin-4-yl)methyl)-3-phenoxybenzamide (TP-064) provided structural evidence for the specificity of inhibition. We also showed that TP-064 inhibited the growth of a subset of multiple myeloma (MM) cell lines. These results indicate that TP-064 may be an effective drug for the treatment of MM that acts by targeting PRMT4.

## RESULTS AND DISCUSSION

### Identification and characterization of the selective PRMT4 inhibitor TP-064

**Figure 1:**
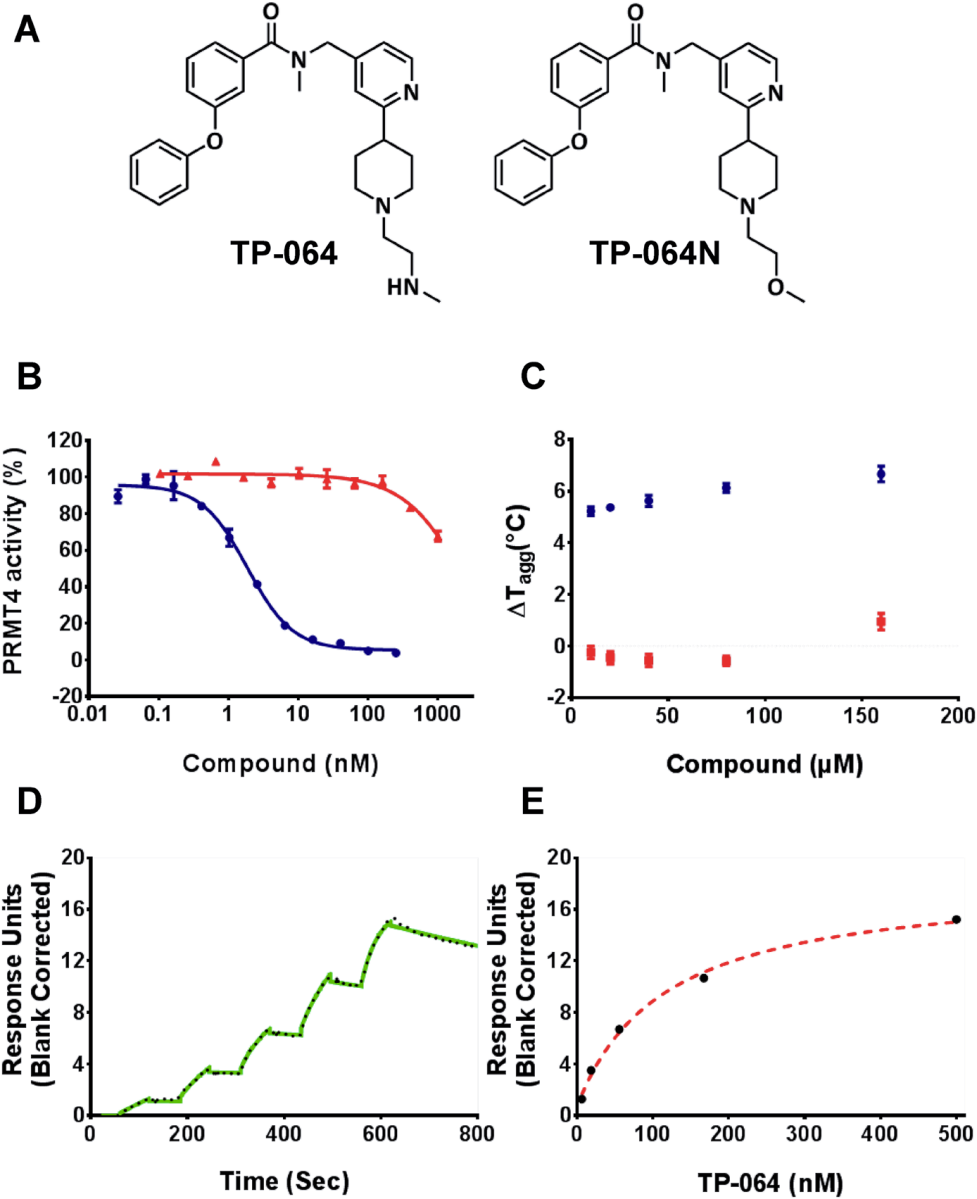
TP-064 is a potent inhibitor of PRMT4 **(A)** Chemical structure of TP-064 and its negative control, TP-064N. **(B)** TP-064 (blue) inhibits PRMT4 activity with an IC_50_ value of < 10 nM under balanced conditions. TP-064N (red) has no effect on PRMT4 activity up to 100 nM. **(C)** The binding of TP-064 to PRMT4 was confirmed by DSLS with stabilization at about 6°C. No binding was observed with TP-064N. **(D, E)** SPR analysis of the TP-064 binding to PRMT4 in the presence of 50 μM SAM. (D) A representative sensorgram (black dots) is shown with the kinetic fit (solid green). A K_d_ value of 7.1 ± 1.8 nM, with k_on_ = 1.1 ± 0.1 × 10^5^ M^−1^ s^−1^ and k_off_ = 0.7 ± 0.1 × 10^−3^ s^−1^, was obtained from triplicate experiments. (E) The steady state response (black circles) and 1:1 binding model fitting (red dashed line) is presented.

TP-064 was developed as a small-molecule inhibitor of PRMT4 (Figure 1A) by chemically optimizing seed compounds identified by high-throughput chemical library screening with a methyltransferase. We found that TP-064 inhibited the methyltransferase activity of PRMT4, with an IC_50_ value of < 10 nM (Figure 1B). The binding of TP-064 to PRMT4 was confirmed by differential static light scattering (DSLS), with aggregation temperature (T_agg_) increasing by about 6°C at 80 μM (Figure 1C). Surface plasmon resonance (SPR) analysis revealed that binding only occurred in the presence of S-adenosyl methionine (SAM), yielding a K_d_ value of 7.1 ± 1.8 nM, with k_on_ = 1.1 ± 0.1 × 10^5^ M^−1^s^−1^ and k_off_ = 0.7 ± 0.1 × 10^−3^ s^−1^ from kinetic fitting (Figure 1D). The steady state response and 1:1 binding model fitting is also presented in Figure 1E. A similar binding profile was also obtained in the presence of S-adenosyl-L-homocysteine (SAH) (Supplementary Figure 1). The observation that the presence of SAM is required for TP-064 binding suggests that SAM binds first, and PRMT4 may follow a similar ordered kinetic mechanism as reported by Brown et. al. for PRMT1 [21]. We found that switching the terminal aminomethyl (TP-064) to a methoxy moiety to obtain N-((2-(1-(2-methoxyethyl)piperidin-4-yl)pyridin-4-yl)methyl)-N-methyl-3-phenoxybenzamide (TP-064N) clearly reduced the inhibitory activity against PRMT4 (IC_50_ 2.5 ± 0.6 μM; Figure 1B). TP-064N binding to PRMT4 was not observed by DSLS (Figure 1C). The high structural similarity and marked difference in potency indicated that TP-064N could serve as a negative control compound for TP-064.

**Figure 2:**
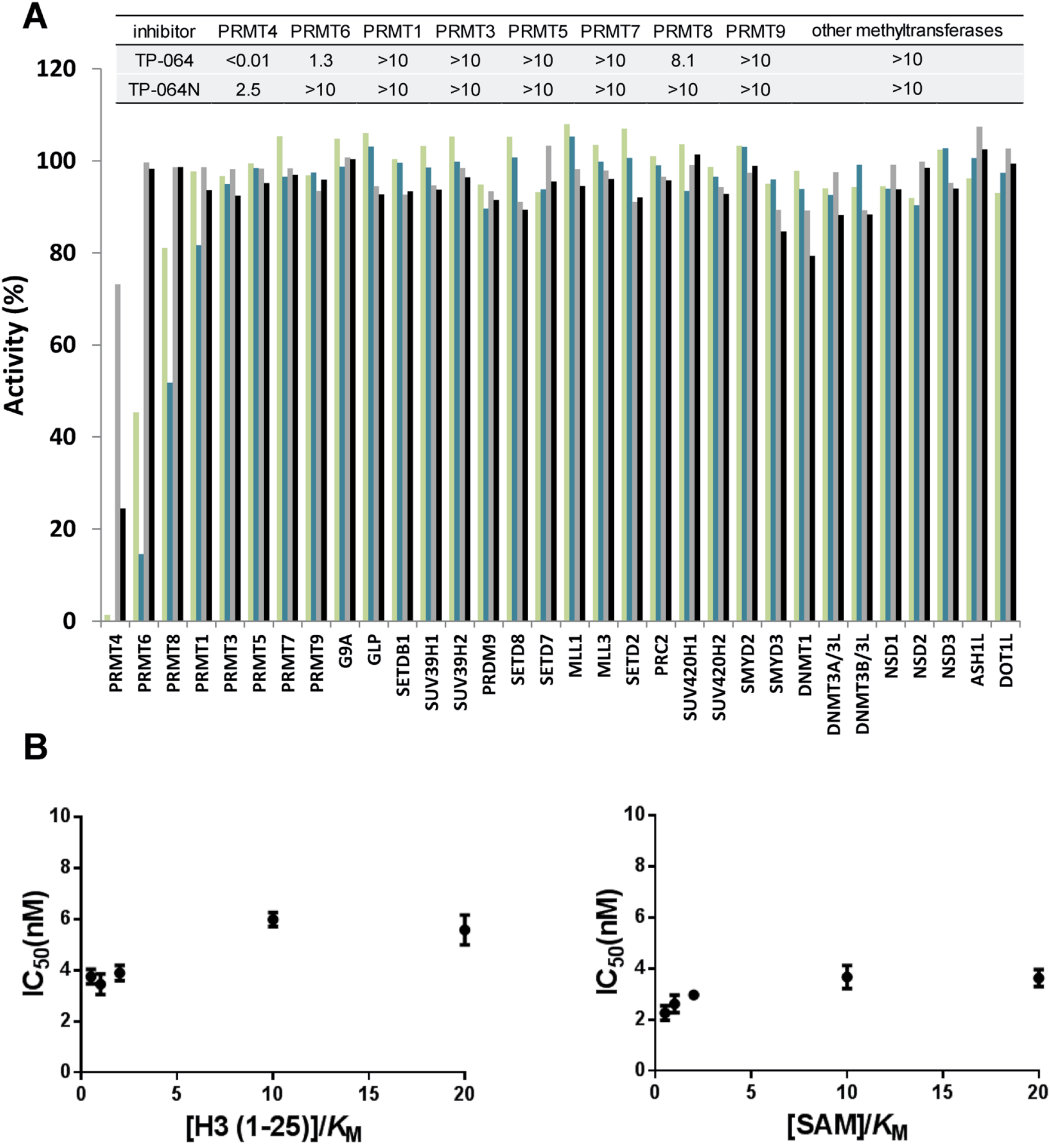
Selectivity and mechanism of action of TP-064 **(A)** Selectivity of TP-064N at 10 μM (■) and 1 μM (■) and of TP-064 at 10 μM (■) and 1 μM (■) for PRMT1, 3, 4, 5, 6, 7, 8, and 9 as well as for 24 histone and DNA methyltransferases was assessed. Dose response data are presented in the top panel as IC_50_s (μM). **(B)** Mechanism of action of TP-064 was assessed by determining IC_50_ of both substrates values at various concentrations.

We evaluated the selectivity of TP-064 against all known human PRMTs except for PRMT2, which was not active in our hands. As summarized in Figure 2A, TP-064 showed high selectivity for PRMT4 over other PRMTs (> 100 fold) (Figure 2A). It was inactive against the other family members (IC_50_ > 10 μM) except for PRMT6 (IC_50_ of 1.3 ± 0.4 μM), which is the most structurally related to PRMT4 among PRMT family [22] and PRMT8 (IC_50_ of 8.1 ± 0.6 μM). To further assess the selectivity of TP-064, we tested it against 24 protein lysine methyltransferases and DNA methyltransferases. TP-064 did not inhibit any of these methyltransferases up to 10 μM (Figure 2A). Negative control compound TP-064N was completely inactive against other methyltransferases (Figure 2A).

To determine the mode of inhibition of TP-064, we investigated the effects of the cofactor SAM and substrate peptide concentration on the IC_50_ values of TP-064 against PRMT4. Increasing SAM or peptide concentration did not significantly affect the IC_50_ values of TP-064 against PRMT4 (Figure 2B), suggesting a non-competitive mode of inhibition, which was previously reported for other inhibitors of methyltransferases including PRMT4 [17] that target the substrate-binding pocket [23, 24].

**Figure 3:**
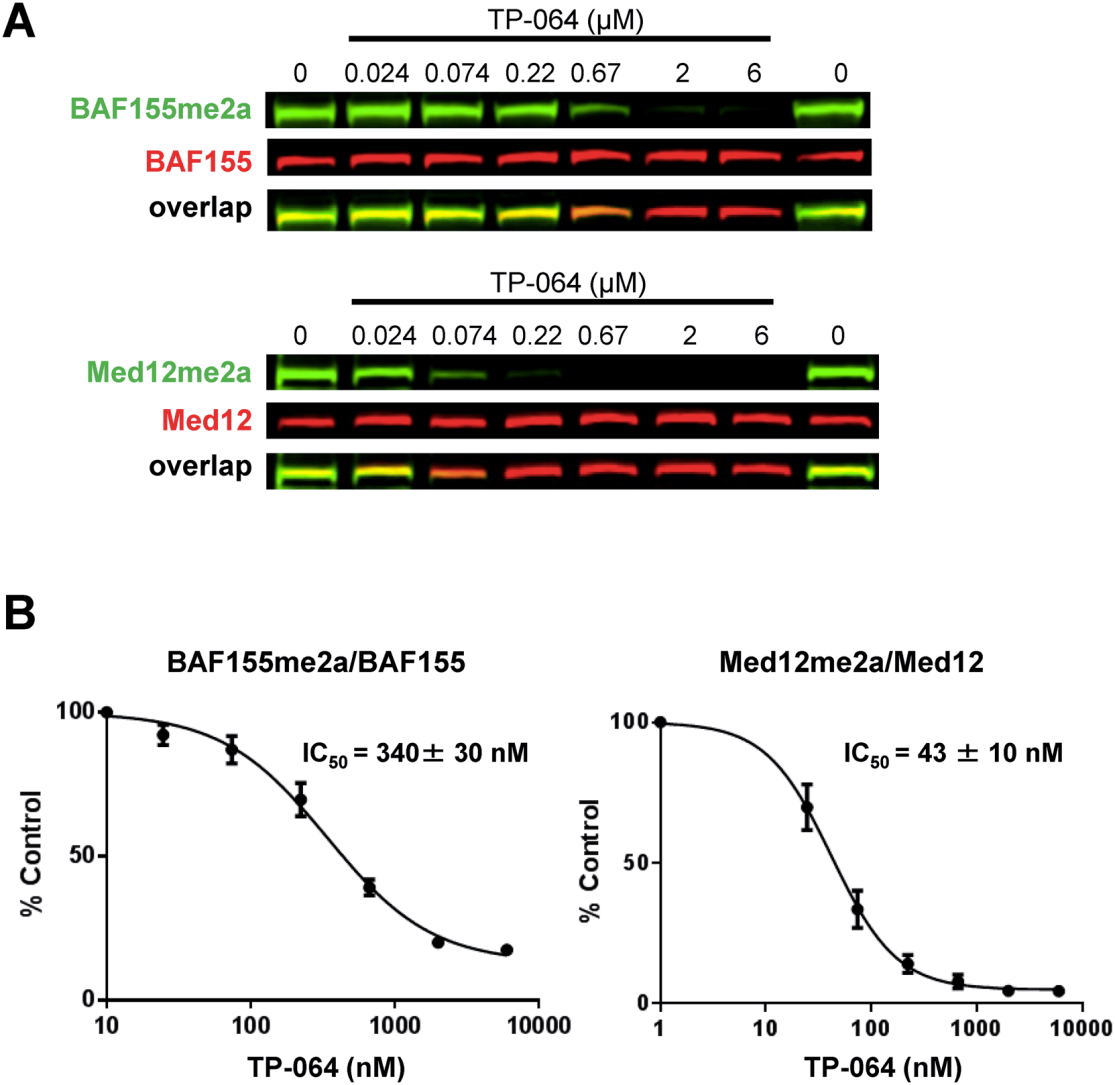
TP-064 inhibits PRMT4 substrate methylation in cells **(A)** TP-064 inhibits the dimethylation of PRMT4 substrates. HEK293 cells were treated with indicated concentrations of TP-064 for 3 days and dimethylation levels of BAF155 and MED12 in whole cell extracts were analyzed by western blotting. **(B)** Quantitation of data in (A). Graphs represent nonlinear curve fits of dimethyl-BAF155 and dimethyl-MED12 signal intensities normalized to total BAF155 or MED12, respectively. Data represent mean ± SEM of two independent experiments prepared in triplicate.

We evaluated the effect of TP-064-mediated inhibition of endogenous PRMT4 in cell-based assays. BRG1-associated factor (BAF)155 and Mediator complex subunit (MED)12 are direct substrates of PRMT4; arginine dimethylation of these proteins is drastically reduced in PRMT4-deficient cells [14, 25]. We found here that TP-064 treatment reduced dimethylation of BAF155 (IC_50_ = 340 ± 30 nM) and MED12 (IC_50_ = 43 ± 10 nM) in a dose-dependent manner (Figure 3A), whereas TP-064N up to concentrations of 10 μM did not inhibit BAF155 and MED12 dimethylation (Supplementary Figure 2). Differences in cellular IC_50_ values for various PRMT4 substrates are not unexpected, as the binding affinities of different substrates could be significantly different as well as the slow substrate turnover rates such as they have been reported for BAF155 complex components [26] could result in higher IC_50_ values. These results indicate that TP-064 is a potent, highly selective, and cell-active PRMT4 inhibitor that can serve as a useful tool for studying the physiological and pathological functions of PRMT4.

### Co-crystal structure of PRMT4 in complex with TP-064

**Figure 4:**
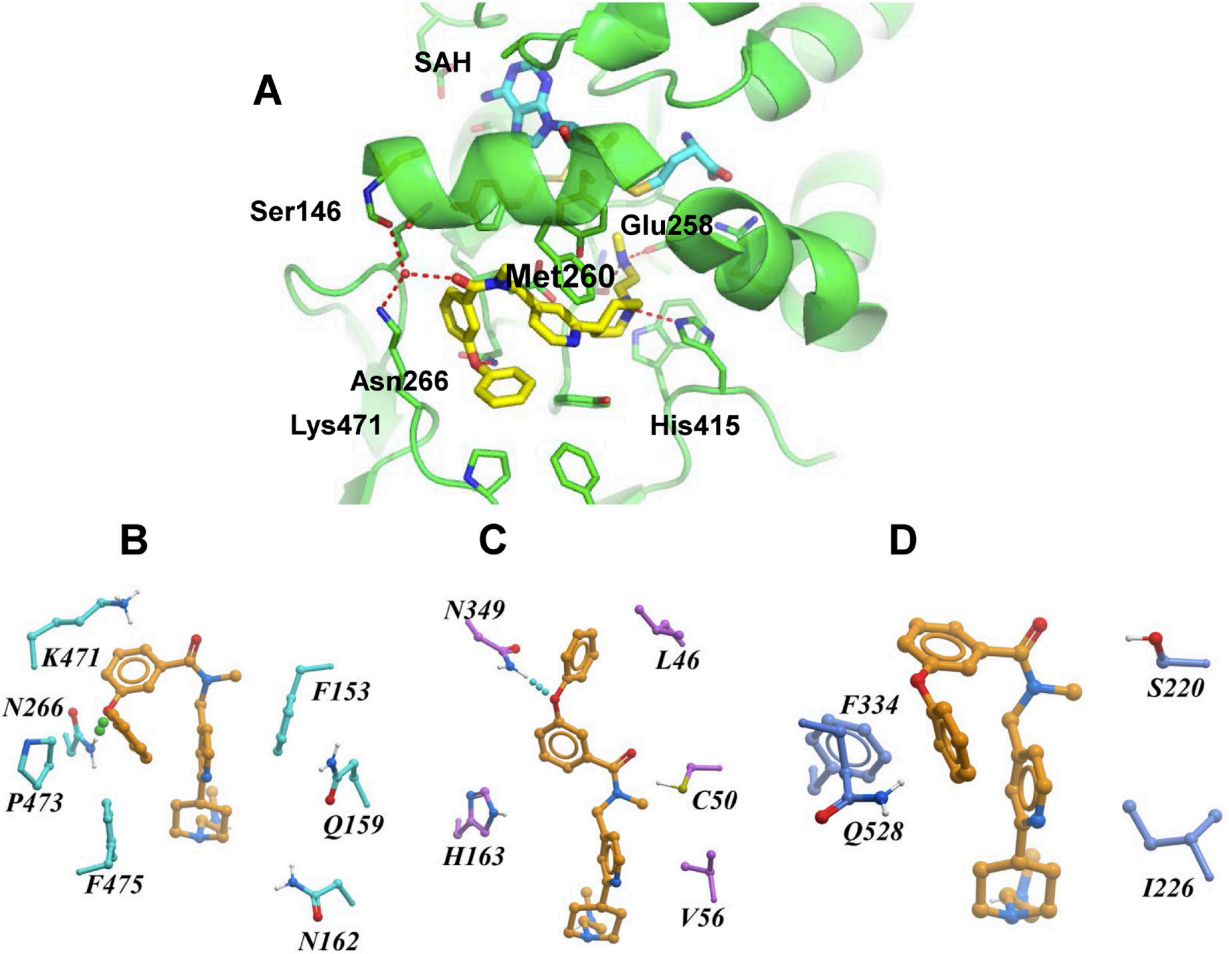
X-ray crystal structure and binding mode of TP-064, SAH, and PRMT4 **(A)** Ribbon diagram of X-ray co-crystal structure of PRMT4 in complex with TP-064 and SAH (PDB code: 5U4X). **(B–D)** Binding mode of TP-064 against PRMT4 (B) and predicted binding mode of TP-064 against PRMT6 (C) and PRMT3 (D) are shown as stick diagrams. Residues in the active site are shown as cyan sticks. Dashed lines represent intermolecular hydrogen bonds.

To clarify the molecular mechanism of TP-064 inhibition, we obtained a co-crystal structure of the catalytic domain of human (h)PRMT4 with TP-064 and the cofactor product SAH at 1.88 Å resolution (PDB code 5U4X) (Figure 4A); the crystal diffraction data and refinement statistics are shown in Supplementary Table 1. The structure showed that TP-064 was bound to the substrate-binding site adjacent to SAH. The methylaminoethyl tail of TP-064 occupied the arginine-binding pocket and formed three hydrogen bonds with PRMT4: two were between the side chain of Glu258 and backbone carbonyl group of Met260, and one was between N1 and the side chain of His415, suggesting the importance of this moiety for strong PRMT4 inhibition. The other moieties of TP-064 engaged in hydrogen bonding with the side chain of Asn266 and hydrophobic interactions (Figure 4A).

To determine the structural basis for the observed selectivity of TP-064 for PRMT4 over other PRMTs, we generated a docking model of this compound in complex with PRMT3 and PRMT6 with Glide [27] using the crystal structure of rat (r) PRMT3 (PDB code 1F3L) and hPRMT6 (PDB code 5E8R). In the PRMT4 complex, TP-064 engaged in a π-stacking interaction with Phe153 and formed a hydrogen bond with Asn266 (Figure 4B). The π-stacking was lost with PRMT6 (Figure 4C), and both interactions were lost with PRMT3 (Figure 4D). Hydrophobic interactions with Pro473, Phe475, and water-mediated hydrogen bonding with Lys471 and Ser146 were also observed only with PRMT4. These results were supported by molecular dynamics and molecular mechanics/generalized Born surface area calculations, which showed that the binding energy of TP-064 was stronger with PRMT4 than with PRMT3 and 6 (Supplementary Table 2). These observations suggest that structural features determine the specific inhibition by TP-064 of PRMT4 over other Type I PRMTs.

Although we did not observe a clear pattern of competitive inhibition for TP-064 (Figure 2B), the crystal structure showed that TP-064 occupied the substrate-binding site of PRMT4. This was not unexpected, since a similar pattern was previously reported for inhibitors of PRMT4 [17] and other methyltransferases [23, 24]. It was suggested that in such cases the binding affinity of the peptide substrate is derived from regions outside the arginine-binding pocket. Thus, TP-064 can occupy this pocket without interfering with substrate-PRMT4 binding to form the substrate-TP-064-PRMT4 complex.

### TP-064 inhibits MM cell proliferation

**Figure 5:**
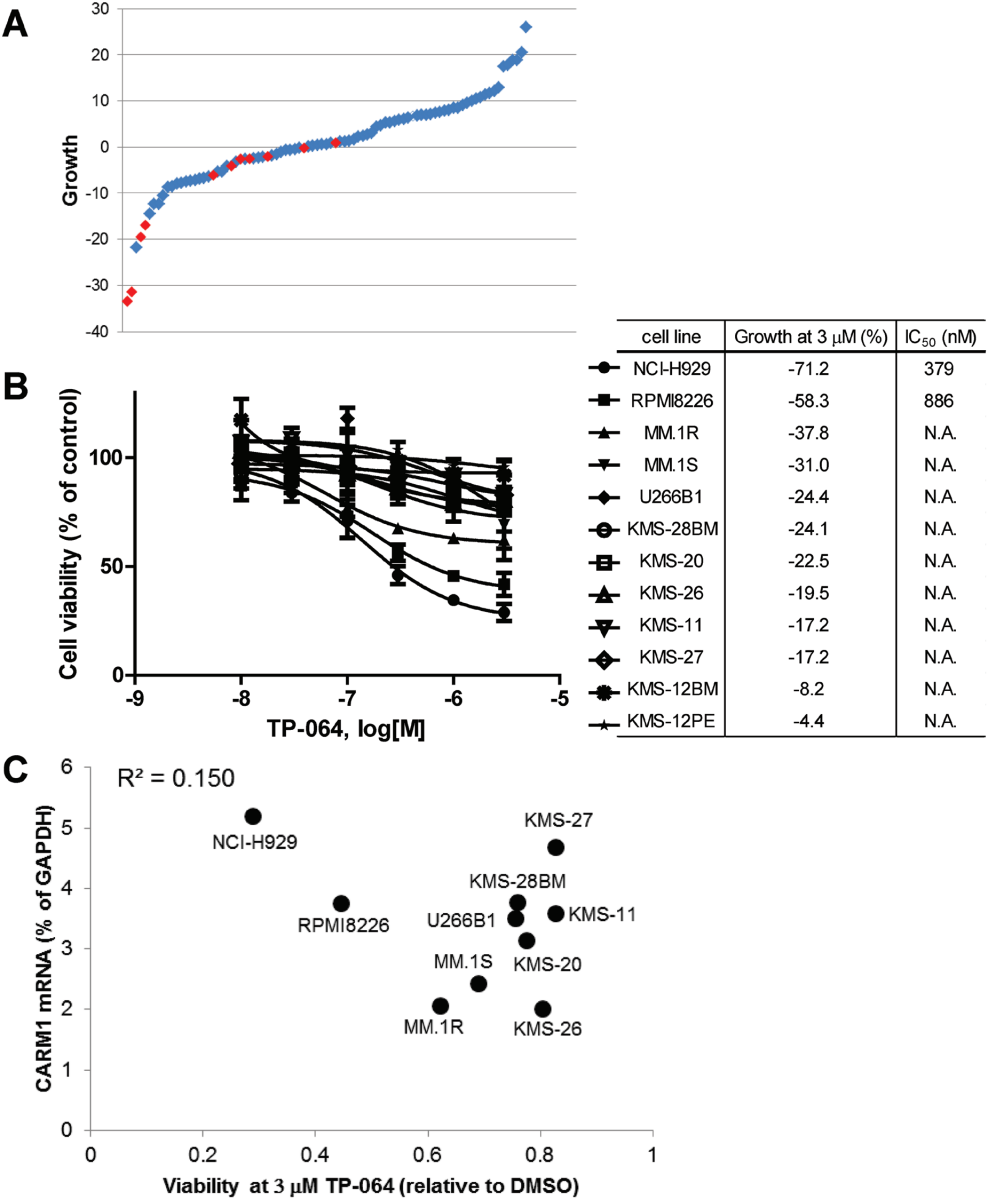
TP-064 inhibits growth in multiple myeloma (MM) cell lines **(A)** A panel of 89 different cancer cell lines was cultured with 3 μM TP-064 for 3 days and cell viability was determined with the CellTiter-Glo assay. % Decrease in viability relative to DMSO-treated cells was defined as growth inhibition and is shown as a water-fall plot (n = 3). Red dots indicate MM cell lines. **(B)** MM cells were treated with TP-064 for 6 days and cell growth was assayed with CellTiter-Glo. Data are presented as mean ± standard deviation (n = 3). IC_50_ values were calculated by nonlinear regression analysis of % inhibition. TP-064 inhibits the growth of NCI-H929, RPMI8226, and MM.1R cells in a dose-dependent manner. **(C)** Correlation between the antiproliferative activity of TP-064 and *PRMT4* mRNA expression in MM cell lines. X and Y axes indicate the relative ATP level at 3 μM TP-064 and *PRMT4* mRNA levels in the 10 indicated MM cell lines, respectively. ATP concentration was calculated based on chemiluminescence values relative to the 0 nM value (control) in each cell line. *PRMT4* mRNA expression levels in MM cells were determined with the Ion Ampliseq transcriptome assay and were normalized to that of glyceraldehyde 3-phosphate dehydrogenase (GAPDH) in each cell line.

To evaluate the effects of TP-064 on cancer cell proliferation, we tested a panel of 89 (69 solid and 20 hematologic) cancer cell lines (Supplementary Table 3). The cells were treated with 3 μM TP-064 for 3 days and viability was evaluated based on intracellular ATP concentration. TP-064 inhibited the growth of a subset of MM cell lines (red dots in Figure 5A). To confirm this result, we carried out a dose titration experiment for a longer treatment period—i.e., 12 MM cell lines were cultured with TP-064 for 6 days. TP-064 treatment inhibited the growth of NCI-H929, RPMI8226, and MM.1R cells in a dose-dependent manner (Figure 5B), but had no effect on acute myeloid leukemia, colon cancer, or lung cancer cell lines (Supplementary Figure 3), suggesting that the efficacy of TP-064 was dependent on the context. As expected, TP-064N did not affect cell growth of MM cells (Supplementary Figure 4). In addition, to further validate the role of PRMT4 in MM cell growth, we performed PRMT4 knockdown in NCI-H929 cells and observed PRMT4 knockdown-induced growth inhibition (Supplementary Figure 5). Thus, pharmacological inhibition of PRMT4 by small molecules may be a therapeutic option for some MM treatment.

To identify a biomarker for predicting the sensitivity of MM cells to TP-064 treatment, we obtained the steady-state transcriptome data of the MM cells used in the growth inhibition assay (GSE110180). At first, we investigated the correlation between sensitivity to TP-064 and *PRMT4* mRNA expression. However, the anti-proliferative effect of TP-064 was not associated with *PRMT4* mRNA levels in the tested cancer cell lines (R^2^ = 0.15; Figure 5C). This indicates that the sensitivity of cancer cells to TP-064 cannot be predicted solely by their expression of PRMT4, and involves other proteins or pathways. Further analysis of the gene expression data in the TP-064 sensitive cells and insensitive cells may shed light on sensitivity markers for the TP-064 treatment in MM cells.

### Pharmacodynamic biomarker inhibition by TP-064 in MM cells

**Figure 6:**
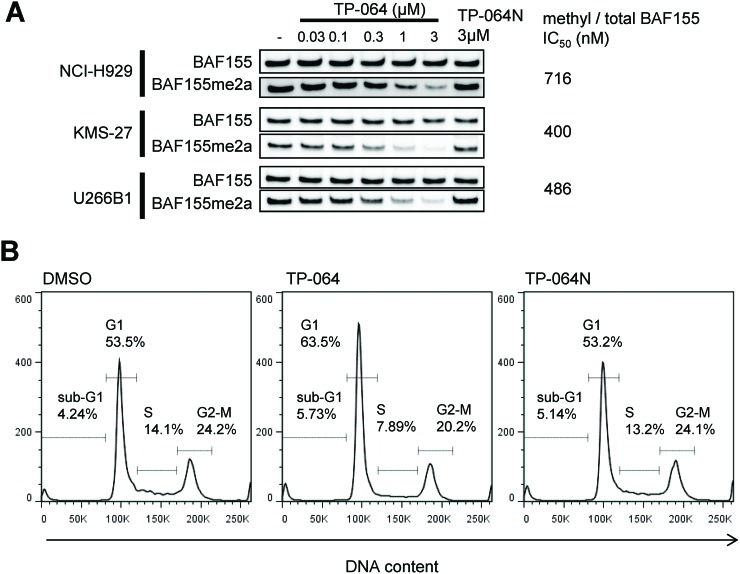
Cellular responses of MM cells treated with TP-064 **(A)** Cells were treated with indicated concentration of TP-064 for 3 days and whole cell extracts were analyzed by western blotting for BAF155 dimethylation. IC_50_ values were calculated by nonlinear regression analysis of % inhibition. **(B)** NCI-H929 cells were treated with DMSO, 1 μM TP-064 or 1μM TP-064N for 72 h, and DNA content was determined by flow cytometry. Sub-G1, G1, S, and G2-M cell fractions are indicated.

To confirm the inhibition of PRMT4 activity in TP-064-sensitive and insensitive MM cells, we evaluated the dimethylation level of BAF155 as a pharmacodynamic biomarker upon TP-064 treatment. TP-064-sensitive NCI-H929 and TP-064-insensitive KMS-27 and U266B1 cells were treated with various concentrations of TP-064 or TP-064N for 72 h and cell lysates were evaluated by western blotting to determine the expression and dimethylation levels of BAF155. Dimethyl-BAF155 level was reduced by TP-064 treatment in a dose-dependent manner in both TP-064-sensitive and -insensitive cells (Figure 6A), whereas TP-064N had no effect. The fact that the observed reduction by TP-064 was not correlated with TP-064 sensitivity suggests that the mechanism of action of TP-064 does not involve BAF155 dimethylation. Although dimethyl-BAF155 cannot be used as a biomarker for predicting TP-064 efficacy, it can nonetheless be used to monitor target inhibition in future pre-clinical and clinical studies of PRMT4 inhibitors.

### TP-064 induces G1 cell cycle arrest in NCI-H929 cells

To clarify the mechanism of TP-064-induced growth inhibition in MM cells, we analyzed cell cycle by flow cytometry. TP-064 treatment reduced the proportion of NCI-H929 cells in S and G2/M phases while increasing the G1 phase fraction (Figure 6B). TP-064N treatment showed no/little effect on cell cycle of the cells. These results imply that PRMT4 inhibition by TP-064 induced G1 cell cycle arrest, although the underlying mechanism remains to be determined. Given that PRMT4 is known to be involved in multiple biological functions and has a wide range of histone and non-histone substrates, comprehensive analyses of the transcriptome, proteome, and methylome and chromatin immunoprecipitation sequencing in TP-064-treated cells can provide insight into the regulation of PRMT4-mediated growth and survival in MM cells as well as biomarkers for evaluating the efficacy of PRMT4 inhibitors.

Recently, CRISPR-based genetic screening has revealed a synergistic interaction between PRMT4 and the histone lysine methyltransferase Disruptor of telomeric silencing 1-like (DOT1L) in the K562 chronic myelogenous leukemia cell line [28]. Our preliminary experiments showed that in K562 cells that have no response to DOT1L inhibitor SGC0946 and a weak response to TP-064 but not TP-064N, the combination of DOT1L inhibitor SGC0946 and TP-064 but not TP-064N elicited a stronger cytotoxic response (Supplementary Figure 6), suggesting that PRMT4 can be combined with other agents for cancer treatment.

## MATERIALS AND METHODS

### Reagents

TP-064 and TP-064N were synthesized by Takeda Pharmaceutical Company (Kanagawa, Japan). The procedure for the synthesis of these compounds is described in the Supplementary methods. [^3^H]SAM was purchased from PerkinElmer Life Sciences (Waltham, MA, USA; cat. no. NET155V001MC; specific activity range: 12–18 Ci/mmol). SAM was obtained from AK Scientific (Union City, CA, USA). Biotinylated peptide substrates were purchased from Tufts University Peptide Synthesis Core Facility (Boston, MA, USA).

### Enzymatic assays for PRMTs

The assay conditions and protein constructs used in this study are presented in Supplementary Tables 4 and 5, respectively. The protein purification procedures have been previously described [17, 23, 29, 30]. In the scintillation proximity assay (SPA), specific amounts of enzyme and biotinylated peptide substrate were mixed with the compound and the reaction was initiated by adding SAM. IC_50_ values were measured at the apparent *K*_m_ concentrations of the substrate and SAM. The reaction was quenched by adding an equal volume of 7.5 M guanidine hydrochloride, and the reaction product was measured by SPA using FlashPlate Plus and TopCount NXT HTS plate reader (both from Perkin Elmer Life Sciences).

### Selectivity assay

The selectivity assay was performed as previously described [31]. The effect of test compounds on the methyltransferase activities of Euchromatic histone-lysine N-methyltransferase 2 (G9a), G9a-like protein (GLP), Suppressor of variegation 3-9 homolog (SUV39H)1, SUV39H2, SUV420H1, SUV420H2, SET domain (SETD)2, SETD8, SETD bifurcated 1 (SETDB1), SETD7, Mixed-lineage leukemia (MLL)1 trimeric complex, MLL3 pentameric complex, Enhancer of zeste homolog (EZH)2 trimeric complex, PRMT1, PRMT3, PRMT4, PRMT5/Methylosome protein (MEP)50 complex, PRMT6, PRMT7, PRMT8, PRMT9, PR domain zinc finger protein (PRDM)9, SET and MYND domain-containing (SMYD)2, SMYD3, and DNA methyltransferase (DNMT)1 was assessed by monitoring the incorporation of a tritium-labeled methyl group into substrates with the scintillation proximity assay [32]. Briefly, a 10-μl reaction containing ^3^H-SAM and substrate at concentrations close to the apparent *K*_m_ values for each enzyme was prepared. Two concentrations (1 and 10 μM) of compound were tested. The reactions were quenched with 10 μl of 7.5 M guanidine hydrochloride; 180 μl of 20 mM Tris buffer (pH 8.0) were added, and the mixture was transferred to a 96-well FlashPlate followed by incubation for 1 h. The counts per minute (CPM) was measured on a TopCount plate reader; the CPM in the absence of compound or enzyme was defined as 100% activity and background (0%), respectively, for each dataset.

For DNMT1, the double-stranded DNA substrate was prepared by annealing two complementary strands (biotinylated forward strand: B-GAGCCCGTAAGCCCGTTCAGGTCG and reverse strand: CGACCTGAACGGGCTTACGGGCTC) that were synthesized by Eurofins MWG Operon (Louisville, KY, USA). A filter-based assay was used for DOT1L, Nuclear receptor binding SET domain protein (NSD)1, NSD2, NSD3, ASH1-like histone lysine methyltransferase (ASH1L), DNMT3A/3L, and DNMT3B/3L in which 10 μl of reaction mixture were incubated at 23°C for 1 h, followed by addition of 50 μl of 10% trichloroacetic acid (TCA). The mixture was transferred to filter plates (Millipore, Billerica, MA, USA) that were centrifuged at 2000 rpm (Allegra X-15R; Beckman Coulter, Brea, CA, USA) for 2 min, washed twice with 10% TCA and once with ethanol (180 μl), and centrifuged. After drying, 100 μl MicroScint-O (Perkin Elmer) was added to each well and the plates were centrifuged to remove the liquid. A 70-μl volume of MicroScint-O was added and the CPM was measured with a TopCount plate reader.

### Substrate competition assays

The substrate competition assays were performed by measuring IC_50_ values at various concentrations of one substrate (e.g., peptide) and at saturating concentrations of the other (e.g., SAM) and vice versa, as previously described [33].

### Orthogonal binding confirmation

SPR experiments were performed using a Biacore T200 system (GE Healthcare, Little Chalfont, UK). Approximately 6500 response units of PRMT4 were amino-coupled onto one flow cell of a CM5 chip according to manufacturer's protocol, while another flow cell was left empty for reference subtraction. SPR analysis was performed in HBS-EP buffer composed of 20 mM HEPES (pH 7.4), 150 mM NaCl, 3 mM EDTA, and 0.05% Tween-20 with 5% dimethylsulfoxide (DMSO), and 50 μM SAH. TP-064 was prepared at concentrations of 500, 125, 31.3, and 7.8 nM by serial dilution. Kinetic determination experiments were performed at 20°C using single cycle kinetics with an on time of 180 s, off time of 300 s, and a flow rate of 100 μl min^−1^. To favor complete dissociation of the compound for the subsequent cycle, HBS-EP with 5% DMSO and no SAH was run for 300 s at 50 μl min^−1^, and two blank cycles were run between each cycle. Kinetic curve fitting and *K*_D_ calculations were performed with a 1:1 binding model using Biacore T200 Evaluation software. DSLS was performed as previously described [34] using 100 mM HEPES (pH 7.5), 150 mM NaCl, 3% DMSO, and 0.4 mg ml^−1^ (6 μM) protein in a 10-μl reaction volume.

### Crystallization, data collection, and structure determination

A DNA fragment encoding the methyltransferase domain of human PRMT4 (residues 140–480) was cloned into a baculovirus expression vector pFBOH-MHL (http://www.thesgc.org/sites/default/files/toronto_vectors/pFBOH-MHL.pdf). The protein was expressed in Sf9 cells as an N-terminal hexa-His tag fusion protein and purified by metal chelating affinity chromatography (TALON resin; Clontech, Mountain View, CA, USA) followed by size-exclusion chromatography (Superdex 200; GE Healthcare). Pooled fractions containing PRMT4 were subjected to tobacco etch virus treatment to remove the His-tag. The protein was purified to homogeneity by ion-exchange chromatography.

Purified PRMT4 (6.1 mg ml^−1^) was mixed with TP-064 at a 1:5 molar ratio of protein:inhibitor and crystallized with the sitting drop vapor diffusion method at 20°C by mixing 2 μl of protein solution with 1 μl of the reservoir solution containing 20% PEG3350, 0.2 M ammonium sulfate, and 0.1 M Tris-HCl (pH 8.5).

X-ray diffraction data for PRMT4 + TP-064 were collected at 100 K on a Rigaku FR-E superbright X-ray generator. Data were processed using the HKL-3000 suite [35]. The structure of PRMT4 + TP-064 was isomorphous to PDB entry 4IKP, which was used as a starting model. REFMAC [36] was used for structure refinement. Geometric restraints for compound refinement were prepared with GRADE v.1.102 developed at Global Phasing Ltd. (Cambridge, UK). The COOT graphics program [37] was used for model building and visualization, and MOLPROBITY [38] was used for structure validation.

### Docking

The X-ray crystal structure of rPRMT3 (PDB 1F3L) [39] and human PRMT6 (PDB 5E8R) [18] were used according to a previously described docking protocol [17]. Docking calculations were performed using Glide SP (Schrodinger, NY, USA) with default settings. Hydrogen bonding constraints were imposed with Glu326 and His476 of PRMT3 and Glu155 and His317 of PRMT6.

### Cell culture

786-O, A498, A549, A704, AML-193, C2BBe1, Caki-1, Caki-2, CAMA-1, Caov-3, Caov-4, COLO-205, DMS114, DMS53, G-401, HCT116, HCT15, HCT-8, HPAC, HPAF-II, Hs 746T, HT1080, HT-29, JEG-3, KATOIII, LS174T, MDA-MB-231, MDA-MB-361, MDA-MB-468, MG-63, MIA-PaCa-2, MM.1R, MM.1S, MSTO-211H, NCI-H1755, NCI-H2228, NCI-H226, NCI-H23, NCI-H2452, NCI-H28, NCI-H460, NCI-H520, NCI-H522, NCI-H661, NCI-H69, NCI-H810, NCI-H929, RKO, SK-MEL-2, SK-MEL-24, SK-MEL-28, SK-MEL-5, SKOV-3, SW1271, SW1417, SW620, SW780, SW948, T-24, T47D, T84, TF-1a, THP-1, U266B1, and U2OS cell lines were purchased from American Type Culture Collection (Manassas, VA, USA). CMK-11-5, COLO201, COLO320 DM, Daudi, Kasumi-1, KMM-1, KMS-11, KMS-12-BM, KMS-12-PE, KMS-20, KMS-26, KMS-27, KMS-28BM, KYSE70, MCF-7, MOLM-16, PL-21, RPMI8226, and SKNO-1 cell lines were from the Japanese Collection of Research Bioresources Cell Bank (Osaka, Japan). The A2780 cell line was from DS Pharma Biomedical (Osaka, Japan). The CACO-2 cell line was from RIKEN BioResource Center (Tsukuba, Japan). OVCAR-4 and SF268 cell lines were from National Cancer Institute (Bethesda, MD, USA). The PA-TU-8902 cell line was from Creative Bioarray (Shirley, NY, USA). SW48 cell line was from Horizon Discovery (Cambridge, UK). The OCI-Ly19 cell line was a gift from Dr. Louis Staudt (National Cancer Institute). The HEK293T cell line was a gift from Sam Benchimol, York University. Supplementary Table 3 summarizes the source and culture conditions of each cell line.

### Western blotting

HEK293 cells were lysed in lysis buffer composed of 20 mM Tris-HCl (pH 8.0), 150 mM NaCl, 1 mM EDTA, 10 mM MgCl_2_, 0.5% TritonX-100, and 12.5 U ml^−1^ benzonase (Sigma-Aldrich, St. Louis, MO, USA) and containing complete EDTA-free protease inhibitor cocktail (Roche Diagnostics, Indianapolis, IN, USA). After incubation for 3 min at room temperature, sodium dodecyl sulfate (SDS) was added to a final concentration of 1%. Cell lysates were resolved on 4%–12% Bis-Tris protein gels (Invitrogen, Carlsbad, CA, USA) with MOPS buffer (Invitrogen) and transferred for 1.5 h (80 V) onto a polyvinylidene difluoride membrane (Millipore) in Tris-glycine transfer buffer containing 20% MeOH and 0.05% SDS. Blots were blocked for 1 h in blocking buffer composed of 5% milk and 0.1% Tween 20 in phosphate-buffered saline (PBS) and then incubated overnight at 4°C in blocking buffer containing primary antibodies against MED12 (1:1000) (Abnova, Taipei, Taiwan; H00009968-A01), MED12 with asymmetrically dimethylated arginine (1:1000; gift from Dr. Mark Bedford and Cell Signaling Technology, Danvers, MA, USA), BAF155 (1:500) (Santa Cruz Biotechnology, Santa Cruz, CA, USA; sc32763), or R1064-dimethylated BAF155 (1:2000) (Millipore; ABE1339). After five washes with 0.1% Tween 20 in PBS, the blots were incubated with goat-anti rabbit (IR800-conjugated; 926-32211) and donkey anti-mouse (IR 680; 926-68072) antibodies (both 1:5000 and from LI-COR Biosciences, Lincoln, NE, USA) in Odyssey blocking buffer (LI-COR Biosciences) for 1 h at room temperature, and washed five times with 0.1% Tween 20 in PBS. The signal was detected on an Odyssey scanner (LI-COR Biosciences) at 800 and 700 nm.

Cultured MM cells were harvested and lysed in ice-cold SDS lysis buffer composed of 62.5 mM Tris-HCl (pH 7.5), 1% SDS, and 10% glycerin. The lysates were separated by SDS-polyacrylamide gel electrophoresis and transferred to a nitrocellulose membrane using an iBlot Transfer Stack and iBlot Gel Transfer Device (Thermo Fisher Scientific, Waltham, MA, USA). After incubation with StartingBlock T20 PBS blocking buffer (Pierce, Rockford, IL, USA), the membrane was incubated overnight at 4°C with primary antibodies against BAF155 (1:1000) (Cell Signaling Technology; 11956) and dimethyl-BAF155 (1:1000) (Millipore; ABE1339) in Can Get Signal solution 1 (Toyobo Life Science, Osaka, Japan). After five washes with 0.1% Tween 20 in PBS, the blots were incubated with horseradish peroxidase-conjugated anti-rabbit IgG (1:5000) (Cell Signaling Technology; 7074) in Can Get Signal solution 2 (Toyobo Life Science) for 30 min at room temperature and washed five times with 0.1% Tween 20 in PBS. The membrane was incubated with ImmunoStar LD (Wako Pure Chemical Industries, Osaka, Japan), and signals were detected with an ImageQuant LAS-3000 imaging system (Fujifilm, Tokyo, Japan).

### Cell proliferation assay

Cells were seeded in tissue culture plates and TP-064 was added immediately or after 24 h (described in Supplementary Table 3). After 72 or 144 h, cell viability was evaluated based on intracellular ATP concentrations using the CellTiter-Glo luminescent cell viability kit (Promega, Madison, WI, USA). Chemiluminescence was measured with a microplate reader. IC_50_ values were calculated by 4-parameter logistic regression using GraphPad Prism software. K562 cells were cultured in RPMI 10% FBS. Cell viability was determined on MACSquant (Miltenyi) flow cytometer by SytoxBlue (Invitrogen) dye exclusion. The drug response effects were calculated by using GraphPad Prism software fractional response calculations.

### siRNA transfection

The following siRNAs were obtained from Thermo Fisher Scientific: non-silencing (Silencer negative control siRNA#2, AM4637), siPRMT4#1 (Silencer select CARM1, s20579), siPRMT4#2 (Silencer select CARM1, s20577). siRNAs were transfected into cells using GenomeONE-Si (Ishihara Sangyo) following the manufacturer's protocol. Statistical comparisons were carried out using the Aspin–Welch's t-test.

### Quantitative reverse transcription–polymerase chain reaction (RT-PCR) analysis

Following the designated treatment, total RNA was isolated from cells and purified using an RNeasy Mini Kit (Qiagen). Reverse transcription reactions were performed using a Verso cDNA synthesis kit (ThermoFisher Scientific). Quantitative real-time PCR analysis was performed with a ViiA7 system (Applied Biosystems, Foster City, CA) and TaqMan Fast Advanced Master Mix with TaqMan probes against indicated genes (Applied Biosystems). The 2–ΔΔCt method was applied to analyze the data, using GAPDH mRNA expression as an internal control. The normalized abundance of target mRNAs was expressed relative to the corresponding value for cells treated with non-silencing siRNAs. The following TaqMan probes were used for quantitative RT-PCR analysis: PRMT4 (Hs1092577_m1, Thermo Fisher Scientific), GAPDH (4333764T, Thermo Fisher Scientific).

### Ion ampliseq transcriptome analysis

Total RNA was isolated and purified from KMS-11, KMS-20, KMS-26, KMS-27, KMS-28BM, MM.1R, MM.1S, NCI-H929, RPMI8226, and U266B1 cells using an RNeasy Mini kit (Qiagen, Valencia, CA, USA). A total of 10 ng RNA was reverse transcribed using the Ion AmpliSeq Transcriptome Human Gene Expression kit (Thermo Fisher Scientific) following the manufacturer's protocol. cDNA libraries were amplified and barcoded using Ion AmpliSeq Transcriptome Human Gene Expression core panel and Ion Xpress Barcode Adapter (Thermo Fisher Scientific). The prepared libraries were purified using Agencourt AMPure XP (Beckman Coulter), quantified with the Ion Library TaqMan Quantitation kit (Thermo Fisher Scientific), diluted to 75 pM, and pooled equally. Emulsion PCR, enrichment, and loading were performed on an Ion Chef Instrument. Templated libraries were sequenced on an Ion Proton system using the Ion P1 Hi-Q Chef kit and the Ion P1 Chip kit v.3 (Thermo Fisher Scientific). Ion Proton reads were analyzed using the AmpliSeqRNA analysis plugin (v.5.2.1.2) in Torrent Suite software (Thermo Fisher Scientific). The data are publically available via the NCBI GEO database (GSE110180).

### Cell cycle analysis

To measure DNA content for cell cycle distribution analysis, cells were incubated with 70% ethanol/PBS (v/v) overnight. Fixed cells were stained with propidium iodide and analyzed on a FACS LSRFortessa system (BD Biosciences, Franklin Lakes, NJ, USA).

## CONCLUSION

We identified TP-064, a novel potent, selective, and cell-active PRMT4 inhibitor and its inactive analog TP-064N. TP-064 had anti-proliferative effects in a subset of MM cell lines and potential synergistic activity with another methyltransferase (DOT1L). Our results suggest that small molecule inhibitors of PRMT4 can serve as tools for investigating PRMT4 pharmacology in health and disease and may be used to treat MM and other forms of cancer, including in combination with conventional drugs.

## SUPPLEMENTARY MATERIALS FIGURES AND TABLES





## Abbreviations

PRMT4: protein arginine methyltransferase 4
CARM1: coactivator associated arginine methyltransferase 1
SAM: S-adenosyl-L-methionine
SAH: S-adenosyl-L-homocysteine
MMA: monomethylarginine
SDMA: symmetric dimethylarginine
ADMA: asymmetric dimethylarginine
DSLS: differential static light scattering
SPR: surface plasmon resonance
MM: multiple myeloma
CRISPR: clustered regularly interspaced short palindromic repeats.
