# Supplementary material for: TP-064, a potent and selective small molecule inhibitor of PRMT4 for multiple myeloma
Source: Oncotarget. 2018 Apr 6;9(26):18480–93. doi: 10.18632/oncotarget.24883 (PMC5915086; doi:10.18632/oncotarget.24883)
Supplement: Supplementary file 1 [file oncotarget-09-18480-s001.pdf]

TP-064, a potent and selective small molecule inhibitor of PRMT4 for multiple myeloma

SUPPLEMENTARY MATERIALS

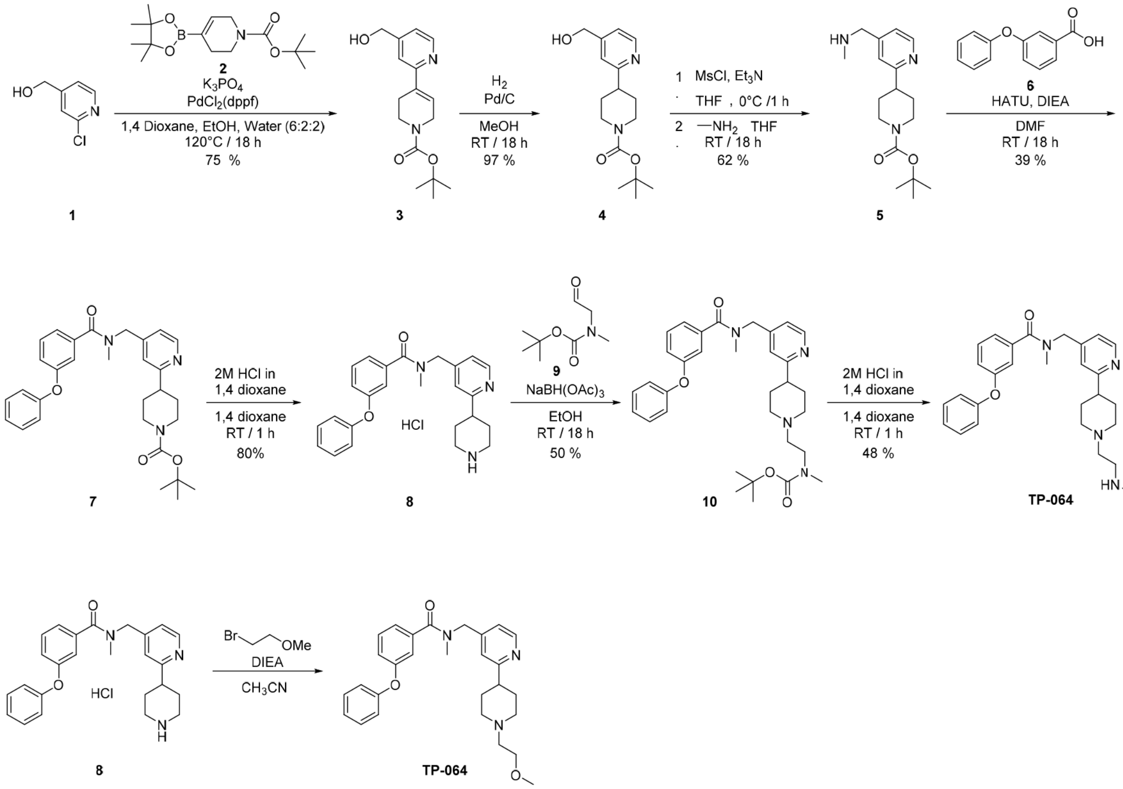

Experimental procedure for 3

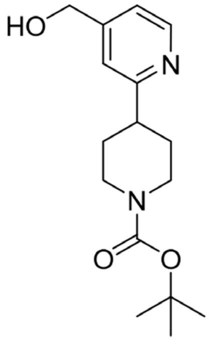

4

Tert-butyl 4-(4-(hydroxymethyl)pyridin-2-yl)-5,6-dihydropyridine-1(2H)-carboxylate

Potassium phosphate (22.2 g, 104.89 mmol, 3 eq.) was added to a solution of (2-chloropyridin-4-yl)methanol (5 g, 34.965 mmol, 1 eq.) and tert-butyl 4-(4,4,5,5-tetramethyl-1,3,2-dioxaborolan-2-yl)-5,6-dihydropyridine-1(2H)-carboxylate (12.97 g, 41.958 mmol, 1.2 eq.) in 1,4 dioxane:ethanol:water (70 ml, 6:2:2) with stirring and argon bubbling for 10 min. This was followed by addition of [1,1bis(diphenylphosphino) ferrocene]-palladium(II) dichloride (1.42 g, 1.748 mmol, 0.05 eq.) in sealed tube under dry atmosphere. The resultant reaction mixture was heated at 120°C for 18 h. The progress of the reaction was monitored by thin-layer chromatography (TLC). After completion of the reaction, the solution was filtered through a celite bed and washed with ethyl acetate; the filtrate was concentrated under reduced vacuum pressure to obtain the crude compound,

which was purified by silica gel (60–120 mesh) column chromatography, eluted with 60% ethyl acetate/pet ether to obtain 3 (7.5 g, yield: 75%) as a yellow solid. The proton nuclear magnetic resonance (^{1}H NMR) (400 MHz, $CDCl_3$) values were as follows: δ 1.48 (9H, s), 2.62-2.64 (2H, m), 3.63 (2H, t, J = 5.38 Hz), 4.09–4.14 (2H, m), 4.74 (2H, s), 6.60 (1H, s), 7.14 (1H, d, *J* = 4.89 Hz), 7.37 (1H, s), 8.50 (1H, d, *J* = 4.89 Hz). Liquid chromatography–mass spectrometry (LC–MS) (M+H): 291.17.

Experimental procedure for 4

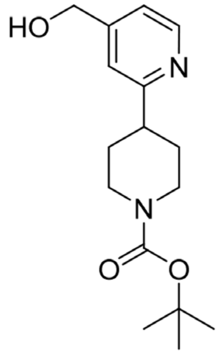

4

Tert-butyl 4-(4-(hydroxymethyl)pyridin-2-yl)piperidine-1-carboxylate

The stirred and degassed solution of **3** (8.5 g, 29.274 mmol, 1 eq.) in methanol (200 ml) was combined with 10% Pd/C (0.311 g, 2.927 mmol, 0.1 eq.) and subjected to hydrogenation under balloon pressure for 18 h. The progress of the reaction was monitored by TLC. After completion of the reaction, the solution was filtered through a celite bed and washed with methanol, and the filtrate was concentrated under reduced vacuum pressure to obtain 4 (7.1 g, yield: 97 %) as an off-white gummy liquid. The ^{1}H NMR (400 MHz, $CDCl_3$) values were as follows δ 1.47 (9H, m), 1.66–1.76 (3H, m), 1.89–1.92 (3H, m), 2.80–2.89 (3H, m), 4.25 (1H, brs), 4.73 (2H, s), 7.12 (1H, d, *J* = 5.38 Hz), 7.16 (1H, s), 8.50 (1H, d, *J* = 4.89 Hz). LC–MS (M+H): 293.21.

Experimental procedure for 5

The stirred solution of 4 (5 g, 17.1 mmol, 1 eq.) in tetrahydrofuran (40 ml) was combined with triethylamine (5.18 g, 51.304 mmol, 3 eq.) and thionyl chloride (2.35 g, 20.52 mmol, 1.2 eq.) at 0°C. The resultant reaction mixture was allowed to stir at room temperature for 1 h before adding 40 ml methylamine (2 M in tetra-n-butylammonium fluoride) at room temperature. The mixture was stirred at room temperature for 18 h, with the progress of the reaction monitored by TLC. After

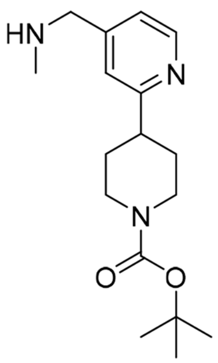

5

Tert-butyl 4-(4-((methylamino)methyl)pyridin-2-yl) piperidine-1-carboxylate

completion of the reaction, the mixture was concentrated under reduced vacuum pressure to obtain the crude compound, which was purified by silica gel (60–120 mesh) column chromatography, eluted with 5% methanol/dichloromethane to obtain 5 (5.09 g, yield: 62 %) as a pale yellow gummy liquid. The ^{1}H NMR (400 MHz, DMSO-d_6) values were as follows: δ 1.41 (9H, s), 1.54–1.58 (2H, m), 1.82 (2H, d, *J* = 10.76 Hz), 2.50 (3H, s), 2.82–2.88 (3H, m), 3.01–3.03 (2H, m), 3.97 (2H, s), 4.02–4.09 (1H, s), 7.22–7.37 (2H, m), 8.50 (1H, d, *J* = 5.38 Hz). LC–MS (M+H): 306.21.

Experimental procedure for 7

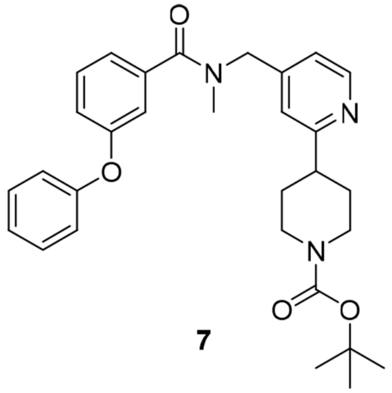

7

Tert-butyl-4-(4-((N-methyl-3-phenoxybenzamido) methyl)pyridin-2-yl)piperidine-1-carboxylate

(1-[Bis(dimethylamino)methylene]-1H-1,2,3-triazolo[4,5-b]pyridinium-3-oxid hexafluorophosphate) (11.4 g, 30.12 mmol, 2 eq.) and N,N-diisopropylethylamine (5.8 g, 45.18 mmol, 3 eq.) were added to the stirred solution of 3-phenoxybenzoic acid (3.87 g, 18.073 mmol, 1.2 eq.) in dimethylformamide (30 ml) at room temperature with stirring for 5 min; 5 (4.6 g, 15.06 mmol, 1 eq.) in dimethylformamide (10 ml) was then added at room temperature with stirring for 18 h.

The progress of the reaction was monitored by TLC. After completion of the reaction, the solution was diluted with water and extracted with ethyl acetate. The Ethyl acetate layer was washed with water and brine solution and dried over anhydrous Na_2SO_4, then filtered and evaporated under reduced pressure to obtain the crude compound. This was purified by silica gel (60–120 mesh) column chromatography, eluted with 60% ethyl acetate/pet ether to obtain compound 7 (2.9 g, Yield: 38.6 %) as a pale yellow gummy liquid. The ^{1}H NMR (300 MHz, $CDCl_3$) values were as follows: δ 1.47 (9H, s), 1.68-1.72 (2H, m), 1.83–1.90 (2H, m), 2.80–3.02 (6H, m), 4.23–4.26 (2H, m), 4.48–4.70 (2H, m), 6.91–7.19 (8H, m), 7.33–7.35 (3H, m), 8.48–8.50 (1H, m). LC–MS (M+H): 502.34.

Experimental procedure for 8

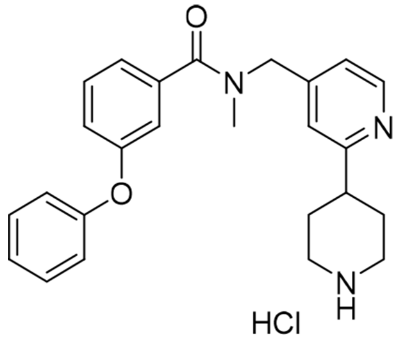

8

N-methyl-3-phenoxy-N-((2-(piperidin-4-yl)pyridin-4-yl)methyl)benzamide hydrochloride

1,4-Dioxane in HCl (2M) (10 ml) was added to the stirred solution of **7** (2.5 g, 5.186 mmol, 1 eq.) in 1,4 dioxane (10 ml) at 0°C and the resultant mixture was stirred at room temperature for 1 h. The progress of the reaction was monitored by TLC. After completion of the reaction, the solution was concentrated under reduced pressure to obtain the crude compound, which was co-distilled with dichloromethane and washed with n-pentane to obtain 8 (1.91 g, yield: 79.5%) as a pale yellow solid. The ^{1}H NMR (300 MHz, DMSO-d_6) values were as follows: δ 1.97–2.06 (4H, m), 2.93–3.02 (5H, m), 3.11–3.18 (1H, m), 3.34–3.38 (2H, m), 4.66 (2H, brs), 6.98–7.01(2H, m), 7.06–7.21 (3H, m), 7.29 (2H, s), 7.35–7.47 (3H, m), 8.51-8.53 (1H, m), 8.82 (1H, brs), 9.38 (1H, brs). LC–MS [(M-2HCl)+H]: 402.25.

Experimental procedure for 10

Sodium triacetoxyborohydride (0.527 g, 2.490 mmol, 5 eq.) was added to the stirred solution of 8 (0.2 g, 0.498 mmol, 1 eq.) and 9 (0.172 g, 0.996 mmol, 2 eq.)

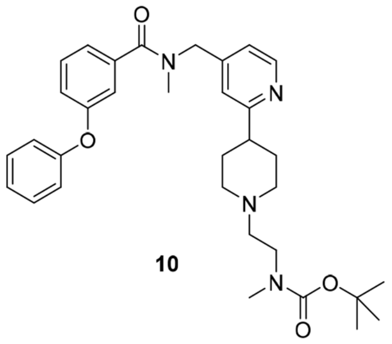

10

Tert-butyl methyl(2-(4-(4-((N-methyl-3-phenoxybenzamido)methyl)pyridin-2-yl)piperidin-1-yl)ethyl)carbamate

in ethanol (20 ml) at 0°C; the resultant reaction mixture was allowed to stir at room temperature for 18 h. The progress of the reaction was monitored by TLC. After completion of the reaction, the solution was basified with saturated sodium bicarbonate solution and extracted with ethyl acetate. The ethyl acetate layer was washed with water and brine solution, dried over anhydrous Na_2SO_4, filtered, and evaporated under reduced pressure to obtain the crude compound, which was purified by silica gel (60–120 mesh) column chromatography and eluted with 3% methanol/ dichloromethane to obtain 10 (0.14 g, yield: 50.3 %) as a brown gummy liquid, which was confirmed by LC–MS. LC–MS (M+H): 559.38.

Experimental procedure for TP-064

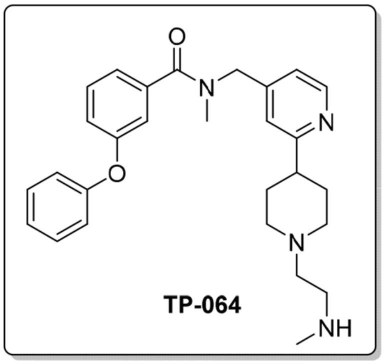

N-methyl-N-((2-(1-(2-(methylamino)ethyl)piperidin-4-yl)pyridin-4-yl)methyl)-3-phenoxybenzamide

1,4-Dioxane in HCl (2M) (2 ml) was added to the stirred solution of 10 (0.14 g, 0.882 mmol, 1 eq.) in 1,4 dioxane (5 ml) was added at 0°C and the resulting reaction mixture was allowed to stir at room temperature for 1 h. The progress of the reaction was monitored by TLC. After completion of the reaction, the reaction mixture was

concentrated under reduced pressure to obtain the crude compound, which was co-distilled with dichloromethane and diethyl ether, basified with triethylamine, and concentrated. The free acid was purified by preparative high-performance LC to obtain TP-064 (55.5 mg, yield: 48.6 %) as a brown semi-solid. The ^{1}H NMR (400 MHz, DMSO-d_6) values were as follows: δ 1.73–1.76 (4H, m), 1.95–2.05 (2H, m), 2.31 (3H, s), 2.40 (2H, t, *J* = 6.26 Hz), 2.60 (3H, t, *J* = 6.26 Hz), 2.88–2.94 (5H, m), 4.46–4.63 (2H, m), 6.94–7.48 (12H, m), 8.43 (1H, brs). LC–MS (M+H): 459.1.

Experimental procedure for TP-064N

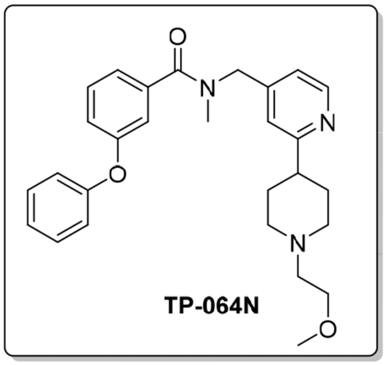

N-((2-(1-(2-methoxyethyl)piperidin-4-yl)pyridin-4-yl)methyl)-N-methyl-3-phenoxybenzamide

A mixture of N-methyl-3-phenoxy-N-((2-(piperidin-4-yl)pyridin-4-yl)methyl)benzamide hydrochloride (2000 mg, 4.57 mmol), 1-bromo-2-methoxyethane (698 mg, 5.02 mmol), and N, N-diisopropylethylamine (3.99 ml, 22.83 mmol) in CH_3CN (15 ml) was stirred at 50°C for 5 h. The mixture was neutralized with saturated $NaHCO_3$ aq. at 0°C and extracted with ethyl acetate. The organic layer was separated, washed with water and brine, dried over $MgSO_4$, and concentrated under vacuum. The residue was purified by column chromatography (NH silica gel, eluted with 50%–100% ethyl acetate in hexane) to obtain N-((2-(1-(2-methoxyethyl)piperidin-4-yl)pyridin-4-yl)methyl)-N-methyl-3-phenoxybenzamide TP-064N (740 mg, 1.610 mmol, 35.3 %) as a white solid. The ^{1}H NMR (300 MHz, DMSO-d_6) values were as follows: δ ppm 1.59–1.84 (m, 4 H) 2.05 (t, *J* = 10.27 Hz, 2 H) 2.43–2.49 (m, 2 H) 2.60 (br. s., 1 H) 2.84–3.03 (m, 5 H) 3.24 (s, 3 H) 3.44 (t, *J* = 5.93 Hz, 2 H) 4.34–4.76 (m, 2 H) 6.82–7.29 (m, 8 H) 7.29–7.61 (m, 3 H) 8.43 (br. s., 1 H). LC–MS (M+H): 460.2.

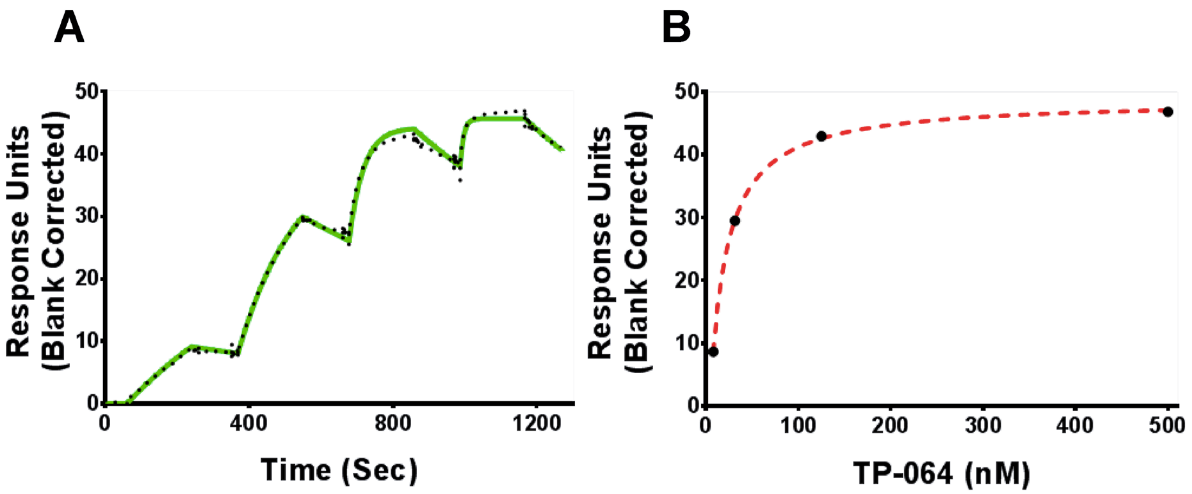

Supplementary Figure 1: SPR analysis of TP-064 binding to PRMT4 in the presence of 50 µM SAH. (A) A representative sensorgram (black dots) is shown with the kinetic fit (solid green). A K_d value of 6.9 ± 1.1 nM, with $k_{on} = 2.02 \pm 0.03 \times 10^5\ M^{-1}\ s^{-1}$ and $k_{off} = 1.4 \pm 0.2 \times 10^{-3}\ s^{-1}$, was obtained from triplicate experiments. **(B)** The steady state response (black circles) and 1:1 binding model fitting (red dashed line) is presented.

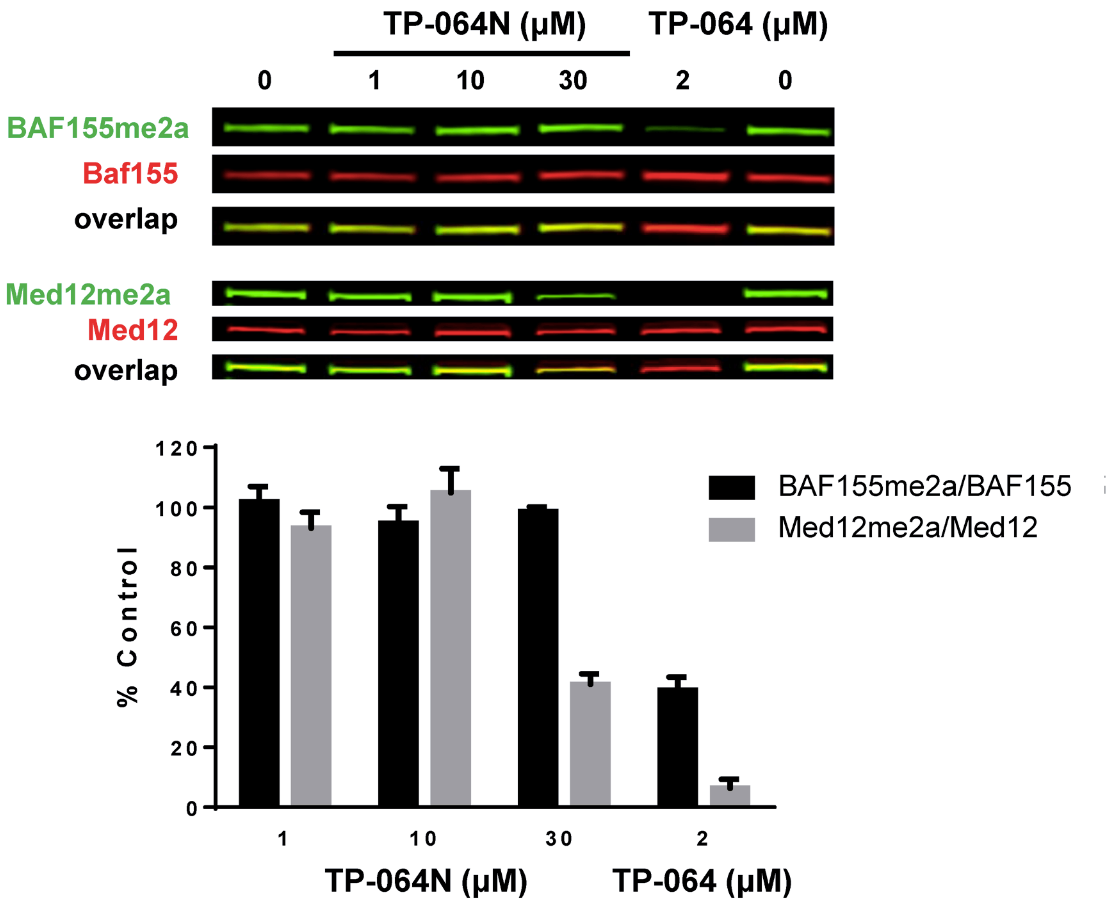

Supplementary Figure 2: Effect of TP-064N on PRMT4 cellular activity. TP-064N did not inhibit the methylation of BAF155 or MED12 up to 10 µM. HEK293 cells were treated with indicated concentration of TP-064 and TP-064N for 3 days and whole cell extracts were analyzed by western blotting for dimethylation of BAF155 R1064 and MED12 R1862.

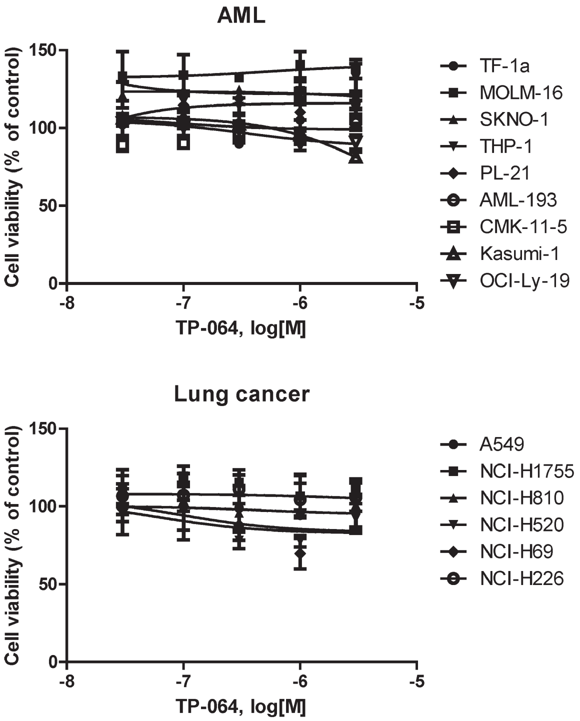

Supplementary Figure 3: Effect of TP-064 on proliferation of various cancer cell lines. TP-064 did not exhibit anti-proliferative activity in acute myeloid leukemia, colon cancer, or lung cancer cell lines treated with indicated concentrations of TP-064 for 6 days. Relative ATP concentration was calculated based on chemiluminescence relative to the 0 nM value (control). Data are presented as mean ± standard deviation (n = 3).

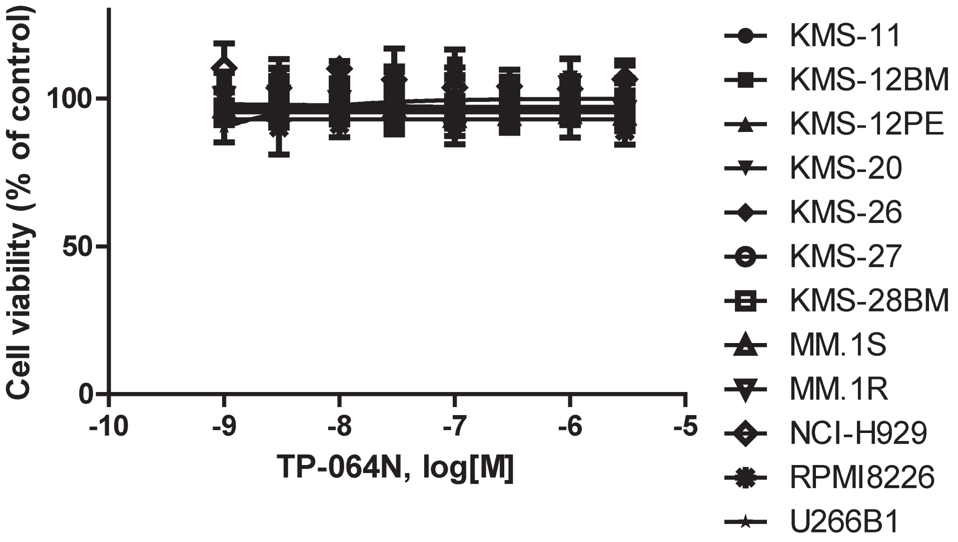

Supplementary Figure 4: TP-064N does not affect the cell growth of MM cell lines. MM cells were treated with indicated concentration of TP-064N for 6 days and cell viability was measured by CellTiter-Glo luminescent cell viability kit. Data are presented as mean ± standard deviation (n = 3).

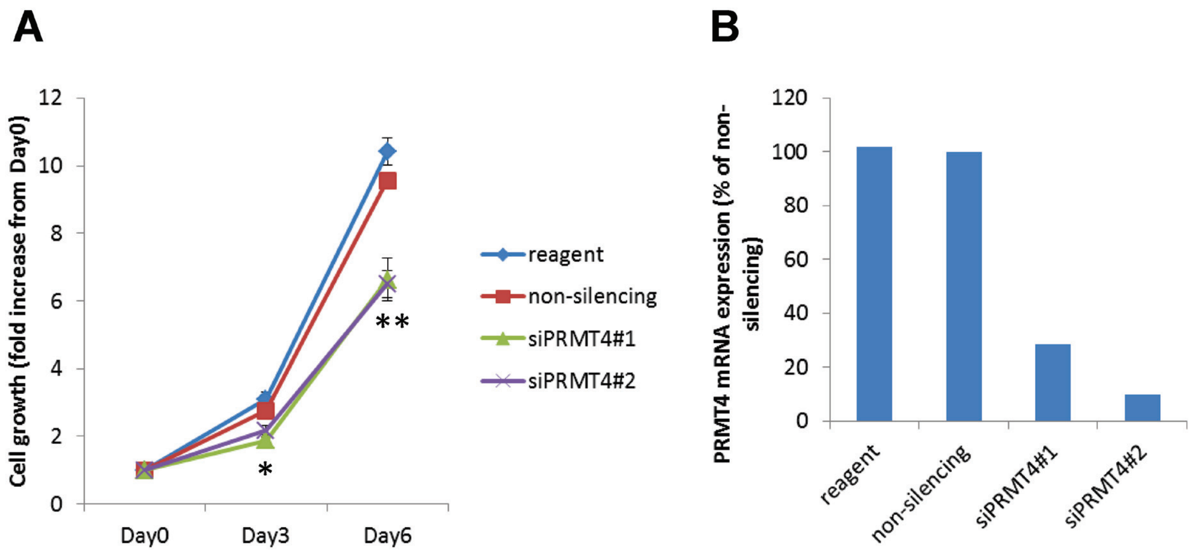

Supplementary Figure 5: PRMT4 knockdown inhibited NCI-H929 cell growth. NCI-H929 cells were transfected with siPRMT4 and cultured indicated period. **(A)** Cell viabilities were evaluated at day 3 and day 6 by CellTiter-Glo luminescent cell viability kit. Data are presented as mean ± SD (n = 4). $^{*}P < 0.01$, $^{**}P < 0.001$, significant differences with the Aspin-Welch's t-test when compared with the values of the non-silencing control. **(B)** Total RNA was isolated from cells at day3 and PRMT4 mRNA expression was evaluated by quantitative RT-PCR.

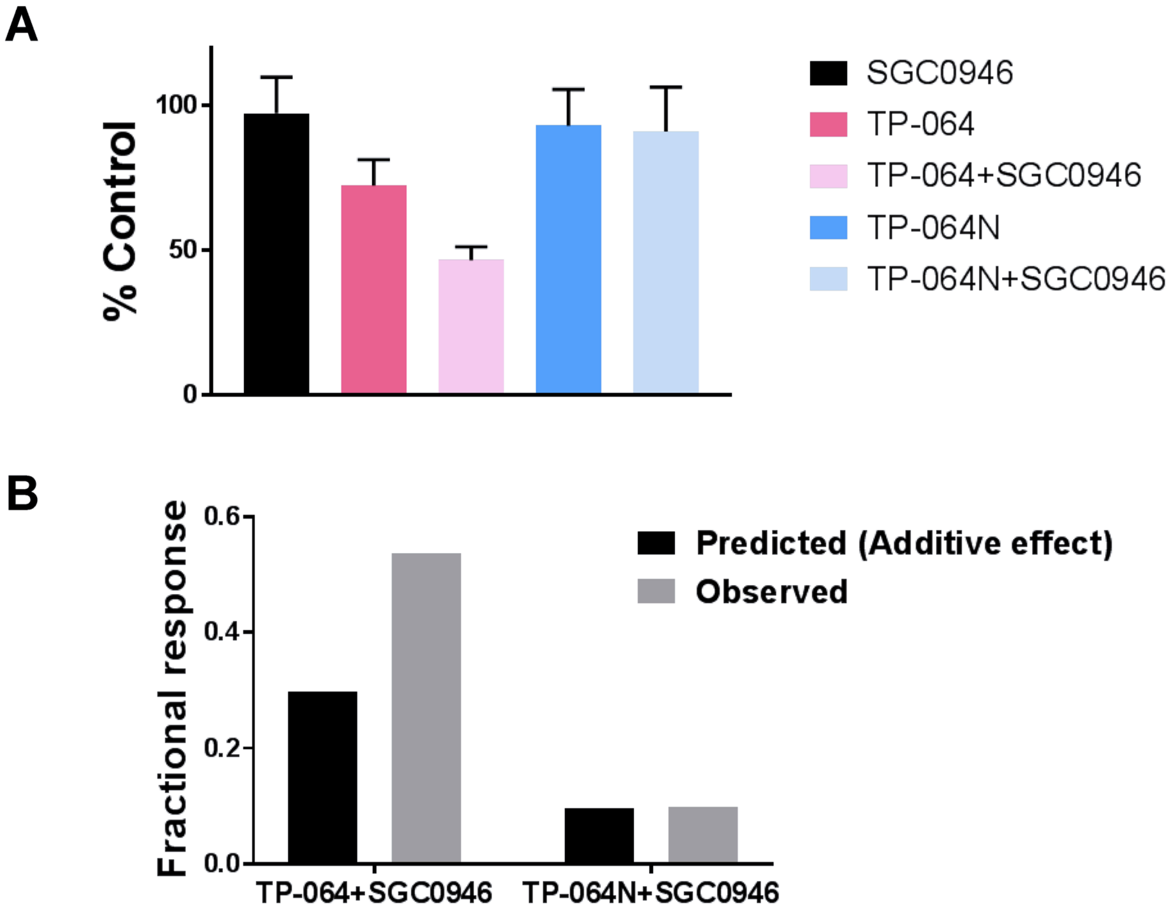

Supplementary Figure 6: Inhibition of DOT1L (SGC0946) and PRMT4 (TP-064) additively suppresses K562 cell growth. K562 cells were treated with 3 µM of TP-064 or TP-064N and 5 µM of SGC0946 for 6 days. The inhibitors were topped up after 3 days. After 6 days, cells were stained with SYTOX Blue dead cell stain and the number of viable cells was determined by flow cytometry. Data are presented as mean ± SD (n = 5). The predicted additive effect was calculated as Fa + Fb × (1 − Fa), where Fa and Fb are the fractional responses to TP-064 and SGC0946, respectively.

Supplementary Table 1: Crystallography data and refinement statistics

	CARM1 + TP-064
**PDB Code**	5U4X
**Data collection**	
Space group	$P2_12_12$
Cell dimensions	
a, b, c (Å)	75.09, 98.92, 207.55
(°)	90.00, 90.00, 90.00
Resolution (Å) (highest resolution shell)	50.00–1.88
	(1.91–1.88)
Unique reflections	126636
$R_{merge(\%)}$	8.9 (102.1)
I/I	22.5
Completeness (%)	99.9 (100.0)
Redundancy	7.7 (7.3)
**Refinement**	
Resolution (Å)	50.00–1.88
No. reflections (test set)	123618 (2521)
$R_{work/}R_{free}$ (%)	21.5/18.8
No. atoms	
Protein	10792
Cofactor	104
Compound	136
Water	700
B-factors (Å^2)	
Protein	28.9
Cofactor	22.6
Compound	25.6
Water	34.4
RMSD	
Bond lengths (Å)	0.009
Bond angles (°)	1.387
Ramachandran plot % residues	
Favored	96.9
Additional allowed	2.8
Generously allowed	0
Disallowed	0.3

Supplementary Table 2: Binding free energy (DG) for TP-064 complexes calculated with the GBSA method*

Complex	G_{GBSA} (Kcal/mol)
PRMT4 + TP-064	−64.6
PRMT3 (1F3L) + TP-064	−54.2
PRMT6 (5E8R) + TP-064	−55

*The protocol for the molecular dynamics simulations in this study are the same as that used in our previous study (https://doi.org/10.1021/acs.jmedchem.6b00668).

GBSA, generalized Born surface area; PRMT3/4/6, protein arginine methyltransferase 3/4/6; TP-064, N-methyl-N-((2-(1-(2-(methylamino)ethyl)piperidin-4-yl)pyridin-4-yl)methyl)-3-phenoxybenzamide.

Supplementary Table 3: Cell lines used in this study

See Supplementary File 1

Supplementary Table 4: Assay conditions for PRMT enzymatic assays

Enzyme	Protein (nM)	Peptide substrate (μM)	^{3}H-SAM (μM)	SAM (μM)	Buffer	Incubation time at 23°C (min)
PRMT1	15	0.13	2	2.6	20 mM Tris-HCl (pH 8.0), 0.01% Triton X-100, 5 mM DTT	45
PRMT3	20	0.57	4	24.3	20 mM Tris-HCl (pH 7.5), 0.01% Tween-20, 5 mM DTT	45
PRMT4	20	0.74	0.5	1.42	20 mM Bicine (pH 8.5), 0.01% Triton X-100	30
PRMT5-MEP50	15	0.07	0.6	0	20 mM Tris-HCl (pH 8.5), 0.01% Tween-20, 5 mM TCEP	30
PRMT6	50	0.6	1.15	1.15	20 mM BTP (pH 7.5), 0.01% Tween-20, 10 mM DTT	30
PRMT7	25	0.3	1.1	0	20 mM Tris-HCl (pH 8.5), 0.01% Tween-20, 5 mM DTT	45
PRMT8	20	0.7	0.5	1.7	20 mM Tris-HCl (pH 8.0), 0.01% Triton X-100, 5 mM DTT	30
PRMT9	10	0.07	5	32.7	20 mM BTP (pH 7.5), 0.01% Tween-20, 10 mM DTT, 0.5 mM EDTA	20

BTP, Bis-Tris-Propane-HCl; DTT, dithiothreitol; EDTA, ethylenediaminetetraacetic acid; MEP50, methylosome protein 50; PRMT, protein arginine methyltransferase; SAM, S-adenosyl-L-methionine; TCEP, (Tris(2-carboxyethyl)phosphine.

Supplementary Table 5: Protein constructs used in PRMT enzymatic assays

Protein	GenBank accession number	Number of amino acids in full-length protein	Amino acids covered
PRMT1	NP_001527.3	371	30–371
PRMT3	XP_011518138.1	426	106–426 (within the identical region of the two isoforms)
PRMT4	NP_954592.1	608	1–608
PRMT5-MEP50	NP_006100.2(PRMT5)	637 (PRMT5)	1–637 (PRMT5)
	NP_077007.1 (MEP50)	342 (MEP50)	1–342 (MEP50)
PRMT6	AAH73866.1	375	1–375
PRMT7	NP_061896.1	692	1–692
PRMT8	AAH22458.2	394	1–394
PRMT9	NP_612373.2	845	1–845

MEP50, methylosome protein 50; PRMT, protein arginine methyltransferase.
